# SGLT2 inhibitors can reduce the incidence of abnormal blood glucose caused by statins in non-diabetes patients with HFrEF after PCI

**DOI:** 10.1186/s12872-023-03353-1

**Published:** 2023-06-27

**Authors:** Yulin Yang, Xiaolin Wang, Yongchao Wang, Hao Xu, Jian Li

**Affiliations:** Department of Cardiology, the Affiliated Hospital of Qingdao University, Qingdao, China

**Keywords:** Abnormal blood glucose, SGLT2 inhibitors, Heart failure, Statins

## Abstract

**Background:**

Taking statins for a long time is associated with an increased risk of new-onset diabetes mellitus. Sodium-glucose cotransporter-2 (SGLT2) inhibitors can reduce insulin resistance and improve pancreatic β-cell function.

**Methods and results:**

In total, 333 non-diabetes patients with heart failure with reduced ejection fraction (HFrEF) after percutaneous coronary intervention (PCI) are included. The enrolled patients are divided into a matched group (n = 198) and an SGLT2 inhibitors group (n = 135). There are no statistical differences in general information between the two groups before treatment. After a mean follow-up time of 13 months, abnormal blood glucose levels are significantly higher in the matched group than in the SGLT2 inhibitors group (6.06 vs. 0.74%, P < 0.05). There are no statistically significant differences in the alanine aminotransferase (ALT), uric acid (UA), and estimated glomerular filtration (eGFR) levels between the two groups.

**Conclusion:**

SGLT2 inhibitors play a significant protective role in reducing the risk of statins-induced abnormal blood glucose in non-diabetes patients with HFrEF after PCI, without increasing the burden on the heart, kidneys, and liver.

## Background

Despite reductions in the prevalence of cardiovascular risk factors and improvements in therapeutic management, coronary artery disease (CHD) remains one of the leading causes of death worldwide [1–3]. In Europe, cardiovascular disease (CVD) causes four million deaths each year, accounting for 45% of all deaths. CHD is the most common cause of CVD-related deaths, accounting for 1.8 million death [4]. CHD is the most common cause of HF and an important therapeutic target for improving HF-associated morbidity and mortality [5]. Statins are the cornerstone of treatment and the prevention of cardiovascular disease. Statins reduce microthromboses and the risk of cardiovascular events [6, 7]. Patients with CHD often need to take statins for a long time, which is associated with a decrease in insulin sensitivity, an increase in fasting plasma glucose levels, and an increased risk of new-onset diabetes mellitus (NODM) [8, 9]. Finding a way to reduce this risk is particularly urgent and important. Sodium-glucose cotransporter-2 (SGLT2) inhibitors reduce the renal reabsorption of glucose and facilitate its urinary excretion by inhibiting the high-capacity glucose transporter SGLT2 in the proximal convoluted tubule, thereby lowering glucose levels independent of insulin [10–12]. SGLT2 inhibitors, a novel class of oral hypoglycemic agents, can reduce insulin resistance and improve pancreatic β-cell function [13]. The Canadian Cardiovascular Society / Canadian Heart Failure Society (CCS/CHFS) Heart Failure Guidelines recommend the use of an SGLT2 inhibitor in patients with HFrEF, with or without concomitant type 2 diabetes, to improve symptoms and quality of life and to reduce the risk of hospitalization due to heart failure (HF) and/or cardiovascular (CV) mortality [14]. It is still unknown whether SGLT2 inhibitors can reduce the risk of statin-induced abnormal blood glucose in non-diabetes patients with HFrEF after PCI. Thus, this study aimed to explore the possibility.

## Methods

### Study design and population

This is a single-center prospective cohor study. A total of 408 patients who underwent PCI at the Affiliated Hospital of Qingdao University between June 2017 and August 2020 are included. The enrolled patients are divided into a matched group (n = 237) and an SGLT2 inhibitors group (n = 171) according to SGLT2 inhibitors use. Loss of follow-up is defined as no documented clinical follow-up for six months. The number of patients lost to follow-up is 39 in the former group and 36 in the latter. Follow-up data are obtained primarily through medical records or telephonic conversations. The inclusion criteria are as follows: (1) patients after PCI; (2) patients diagnosed with HFrEF; and (3) patients on regular statins (including Rosuvastatin, Atorvastatin, Pravastatin and Simvastatin) use. The exclusion criteria are as follows: (1) patients with impaired fasting glucose (IFG), impaired glucose tolerance (IGT), or diabetes mellitus (DM); (2) patients not on regular statin use; (3) patients in whom SGLT2 inhibitors are contraindicated; and (4) patients with severe insufficiency of the liver, kidneys, and other organs. Ethical approval is obtained from the Ethics Committee of the Affiliated Hospital of Qingdao University (QYFYWZLL26346). Informed consent is obtained from all patients. The study is conducted in accordance with the ethical principles of the Declaration of Helsinki.

### Therapies

The matched group is administered conventional treatments, such as antiplatelet agents, lipid-lowering drugs, ACE inhibitors, ARB, beta blockers, diuretics, and aldosterone antagonists. Based on this treatment, the SGLT2 inhibitors group is administered dapagliflozin 5 mg quaque die (QD), or empagliflozin 5 mg quaque die (QD).

### Observational index

The main observational indices include levels of body mass index (BMI), hemoglobin A1c (HbA1c), fasting plasma glucose (FPG), 2 h postprandial blood glucose (2hPG), uric acid (UA), estimated glomerular filtration (eGFR), alanine aminotransferase (ALT), total cholesterol (TC), triglyceride (TG), low-density lipoprotein cholesterol (LDL-C), and left ventricular ejection fraction (LVEF). After 13 months of follow-up, the observational indices of the two groups are measured again. When the observational indices of the SGLT2 inhibitors group are measured, the patients in the SGLT2 inhibitors group have stopped taking the SGLT2 inhibitors for at least 4 months. Adverse reactions, such as hypotension, hypoglycemia, and urinary tract infection, are recorded. The differences in abnormal blood glucose, hypotension, hypoglycemia, and urinary tract infection between the two groups are compared after treatment. And the differences in the ALT, UA, eGFR and ALT levels after treatment are compared.

### Abnormal blood glucose diagnosis

We adopt the World Health Organization (WHO) (1999) criteria for the diagnosis and classification of diabetes. Tables 1 and 2 summarize the classification of metabolic status and diagnostic criteria for diabetes [15]. Impaired fasting glucose is diagnosed based on a fasting plasma glucose concentration greater than or equal to 6.1 mmol/L and less than 7 mmol/L and 2 h postprandial glucose concentration less than 7.8 mmol/L. Impaired glucose tolerance is diagnosed based on a fasting plasma glucose concentration of less than 7 mmol/L and 2 h postprandial glucose concentration greater than or equal to 7.8mmol/L and less than 11.1mmol/L (Table 1). Diabetes is diagnosed based on typical symptoms of diabetes (polydipsia, polyuria, polyphagia, and weight loss) and random plasma glucose concentration greater than or equal to11.1 mmol/L or fasting plasma glucose concentration greater than or equal to 7mmol/L or 2 h postprandial glucose concentration greater than 11.1mmol/L (Table 2). Abnormal blood glucose includes IFG, IGT, and diabetes.

**Table 1:** Categories of abnormal blood glucose

Categories of Hyperglycemia	Venous plasma glucose (mmol/l)	
	FPG	OGTT2hPG
IFG	≥ 6.1, <7.0	<7.8
IGT	<7.0	≥ 7.8, <11.1
Diabetes	≥ 7.0	≥ 11.1

Note. Both IFG and IGT are considered impaired glucose regulation, also known as prediabetes

**Table 2:** Diagnostic criteria for diabetes

1. Typical symptoms of diabetes (polydipsia, polyuria, polyphagia, and weight loss) and random plasma glucose ≥ 11.1 mmol/L
or
2. FPG ≥ 7.0mmol/L
0r
3. OGTT 2hPG ≥ 11.1mmol/L

Note. Fasting was defined as no caloric intake for at least eight hours. Random blood glucose refers to the blood glucose level at any time of the day, regardless of the time of the last meal, which cannot be used to diagnose IFG or IGT. No typical symptoms of diabetes, venous FPG, or OGTT 2Hpg must be retested on different days to confirm diabetes

### Statistical analysis

SPSS (version 26.0; SPSS Inc., Chicago, IL, USA) is used for all statistical analyses. Data are expressed as mean ± standard deviation (SD). Student’s t-test is used to compare the differences between the two groups. The chi-square test is used to evaluate qualitative data. Statistical significance is set at P < 0.05.

## Result

### Patient characteristics

There are no statistical differences in the smoking, drinking, and family diabetic history, age, sex, and levels of MBI, HbA1c, FPG, 2hPG, eGFR, UA, ALT, TC, TG, LDL-C, and LVEF between the two groups before treatment (P > 0.05) (Table 3).

**Table 3:** Comparison of patient characteristics between the two groups before treatment

Variables	Matched group (n = 198)	SGLT2-inhibitor group (n = 135)	t/χ^2^	P -alue
Smoking history	67 (33.84%)	56 (41.48%)	2.01	0.156
Drinking history	59 (29.80%)	33 (24.44%)	1.15	0.283
Diabetic family history	10 (5.05%)	9 (6.67%)	0.39	0.532
Age (years)	60.11 ± 11.73	59.14 ± 9.47	-0.83	0.406
Male	142 (71.72%)	102 (75.56%)	0.60	0.437
MBI (kg•m^− 2^)	26.28 ± 2.42	26.62 ± 2.09	1.36	0.174
HbA1c (%)	5.15 ± 0.60	5.25 ± 0.57	1.63	0.105
FPG (mmol/L)	4.92 ± 0.99	5.04 ± 0.81	1.30	0.195
2hGP/(mmol/L)	7.12 ± 0.44	7.09 ± 0.65	-0.43	0.669
eGFR (ml/min)	102.84 ± 9.40	104.87 ± 13.75	1.49	0.138
UA (µmmol/L)	344.50 ± 52.12	346.67 ± 70.63	0.30	0.761
ALT (U/L)	34.16 ± 24.12	30.66 ± 15.94	-1.60	0.112
TC (mmol/L)	5.05 ± 1.83	5.34 ± 1.39	1.63	0.105
TG (mmol/L)	2.12 ± 1.03	1.97 ± 0.65	-1.62	0.106
LDL-C (mmol/L)	2.58 ± 1.26	2.77 ± 1.20	1.40	0.163
LVEF (%)	36.91 ± 1.93	36.91 ± 1.90	1.30	0.195

Data are represented as n (%) or x ± s. BMI, body mass index; HbA1c, hemoglobin A1c; FPG, fasting plasma glucose; 2hPG, 2 h postprandial blood glucose; eGFR, estimated glomerular filtration rate; UA, uric acid; ALT, alanine aminotransferase; TC, total cholesterol; TG, triglyceride; LDL-C, low-density lipoprotein cholesterol; LVEF, left ventricular ejection fraction

Comparison of general information between the two groups after a mean follow-up of 13 months.

After a mean follow-up time of 13 months, abnormal blood glucose is significantly higher in the matched group (n = 12) than in the SGLT2 inhibitors group (n = 1) (6.06 vs. 0.74%, P < 0.05; RR 0.122, 95% CI: 0.016, 0.929). We conclude that a small dose of SGLT2 inhibitors plays a significant protective role in reducing the risk of statin-induced abnormal blood glucose. There are no statistically significant differences in the ALT, UA, eGER, and ALT levels before and after treatment (P > 0.05) (Table 4).

**Table 4:** Comparison of general information between the two groups before and after treatment

Variables	/Matched group (n = 198)	SGLT2-inhibitor group (n = 135)	t/χ^2^	P-value
Abnormal blood glucose	12 (6.06%)	1 (0.74%)	6.06	0.014
ALT difference (U/L)	3.37 ± 17.32	4.21 ± 9.35	0.58	0.565
UA difference (µmmol/L)	-4.24 ± 57.43	-10.59 ± 27.35	-1.20	0.233
eGER difference (ml/min)	-2.54 ± 7.40	-1.61 ± 4.93	1.37	0.173
Hypotension, hypoglycemia, and urinary tract infection	0(0%)	1(0.74%)		0.405^a^

Data are represented as n (%) or x ± s. Note. a: Fisher’s Exact Test

## Discussion

Statins, 3-Hydroxy-3-methylglutaryl-coenzyme A (HMG-CoA) reductase inhibitors, lower the total cholesterol, low-density lipoprotein, and triglycerides levels, and increase high-density lipoprotein cholesterol levels [16]. Statins are among the main drugs for the primary prevention of atherosclerotic CVD (ASCVD) [17]. The most efficient treatment for atherosclerosis is statins, which decrease the risk of stroke and CHD [18] and reduce CVD-associated morbidity and mortality [19]. However, long-term statin use may reduce insulin sensitivity, increase insulin resistance, and increase the risk of NODM [20–23]. The molecular mechanism of statins-induced abnormal blood glucose is complex and not clearly understood, although several pathophysiological mechanisms have been proposed [24]. It is possibly associated with insulin secretion and resistance alterations, ion channel changes, signaling pathway modulation, oxidative stress, adipocyte differentiation alterations, and leptin and adiponectin modulations [25, 26].

Diabetes is a disease in which blood glucose levels rise abnormally due to absolute or relative insulin deficiency. The three principal diabetogenic factors are adiposity, insulin resistance in skeletal muscle, and decreased insulin production by pancreatic β-cells [27]. In 2012, the FDA added information to labels concerning the effect of statins on incident diabetes and increases in glucose and HbA1c levels [28]. The benefits of statins in preventing CVD events far outweigh the potential risk from plasma glucose level elevation, and thus remain the gold standard for the prevention and management of CVD [29]. SGLT2 inhibitors are a new class of glucose-lowering drugs that reduce glucose reabsorption from the proximal tubule of the kidneys [30–32]. SGLT2 inhibitors not only significantly reduce blood glucose levels but also improve islet beta-cell function and have a protective effect on cardiac muscle cells [33, 34]. Meantime, SGLT2 inhibitors can help control body weight, which is one of three principal diabetogenic factors [27, 35]. Currently, with the publication of the American College of Cardiology Expert Consensus Pathway for patients with HFrEF, SGLT2 inhibitors are included as part of GDMT in patients already on ACEI, ARB or ARNI, beta-blockers, and aldosterone antagonists [36]. This provides a theoretical possibility that SGLT2 inhibitors intervention can reduce the occurrence of statins-induced abnormal blood glucose in non-diabetes patients with HFrEF after PCI. However, whether it can achieve the expected effect remains unknown. This study further explores this issue.

During the 13-month follow-up period, 12 (6.06%) of the 198 patients in the control group developed abnormal blood glucose levels; Seven, four, and one patients developed impaired fasting blood glucose, impaired glucose tolerance, and diabetes, respectively. Among the 135 patients in the SGLT2 inhibitors group, one patient (0.74%) with abnormal blood glucose levels showed impaired glucose tolerance. The rate of abnormal blood glucose is significantly higher in the matched group than in the SGLT2 inhibitors group (6.06 vs. 0.74%, P < 0.05); RR, 0.122, 95% CI: 0.016, 0.929). SGLT2 inhibitors play a significant protective role in reducing the risk of statins-induced abnormal blood glucose in patients with HFrEF after PCI. In this study, the SGLT2 inhibitors group stopped the SGLT2 inhibitors for 4 months before the fasting blood glucose, HbA1c, 2 h blood glucose after glucose load, and other indicators are re-measured. This is done to exclude the influence of SGLT2 inhibitors on the observation indicators. There are no statistically significant differences in the ALT, UA, and eGFR levels, hypoglycemia, low blood pressure, and urinary tract infection (P > 0.05). No electrolyte disturbances are observed in either group. This study innovatively reveals that the application of a small dose of 5 mg SGLT2 inhibitors can reduce the risk of statins-induced abnormal blood glucose in non-diabetes patients with HFrEF after PCI, without increasing the risk of its common side effects.

However, the mechanism by which SGLT2 inhibitors reduce statins-induced abnormal blood glucose in patients with HFrEF after PCI remains unclear. The hypoglycemic effect of the SGLT2 inhibitors itself can cause the islet cells to rest and increase insulin sensitivity. SGLT2 inhibitors may also have a protective effect on pancreatic islets by promoting pancreatic β-cell proliferation and reducing insulin resistance [13]. A study including 456 reports of myopathy with concomitant use of SGLT2 inhibitors and statins finds there is no increased risk of myotoxicity reporting associated with concomitant use of SGLT2 inhibitors and statins[37]. The present findings suggest there is a negative association between the use of SGLT2 inhibitors and the risk of new-onset stroke in patients with type 2 diabetes[38]. Empagliflozin improves endothelial function and reduces mitochondrial oxidative stress[39]. Empagliflozin can increase plasma levels of campesterol and improve the microRNA signature of endothelial dysfunction in patients with HFpEF[40, 41]. In T2DM patients with (acute myocardial infarction) AMI, the use of SGLT2 inhibitors was associated with a lower risk of adverse cardiovascular outcomes during index hospitalization and long-term follow-up[42]. The reduction in the occurrence of abnormal blood glucose may be caused by a combination of the above, single, or multiple mechanisms. More relevant basic research is needed to further explore this issue. The strength of this study is that we innovatively investigate whether the application of SGLT2 inhibitors could reduce the incidence of dysglycemia in patients with coronary artery disease. This study has some shortcomings. The patient’s recent diet may have an impact on the results. In patients with abnormal blood glucose, fasting plasma glucose concentration and 2 h postprandial glucose concentration should be re-measured on the next day to reduce deviations. The patient’s islet function should also be measured. Patients with a family history of diabetes may have some impact on the results. The number of follow-up cases was relatively small. The follow-up time was relatively short. We will gradually increase the number of participants and increase the follow-up period to further improve the reliability of the conclusion. Insulin and C-peptide levels will be added to the ongoing study.

## Conclusion

SGLT2 inhibitors play a protective role in reducing the risk of statins-induced abnormal blood glucose levels in non-diabetes patients with HFrEF after PCI. Additionally, it does not increase the risk of hypotension, hypoglycemia, urinary tract infection, electrolyte disturbance, or the burden on the heart, kidney, and liver.

## Data Availability

The data used to support the findings of this study are available from the corresponding author upon reasonable request.

## Abbreviations

SGLT2: Sodium-glucose cotransporter-2
MBI: body mass index
LVEF: left ventricular ejection fraction
HbA1c: hemoglobin A1c
FPG: fasting plasma glucose
2hPG: 2 h postprandial blood glucose
UA: uric acid
eGFR: estimated glomerular filtration
ALT: alanine aminotransferase
TC: total cholesterol
TG: triglyceride
LDL-C: low-density lipoprotein cholesterol
RR: risk ratio
CHD: coronary artery disease
CVD: cardiovascular disease
NODM: new-onset diabetes
CCS: Canadian Cardiovascular Society
CHFS: Canadian Heart Failure Society
HF: heart failure
CV: cardiovascular
CTA: computed tomographic angiography
CAG: coronary arteriography
PCI: percutaneous coronary intervention
HFrEF: heart failure with reduced ejection fraction
IFG: impaired fasting glucose
IGT: impaired glucose tolerance
DM: diabetes mellitus
ACE: Angiotensin-converting Enzyme
ARB: Angiotensin receptor blocker
QD: quaque die
WHO: World Health Organization
SPSS: statistical product service solutions
USA: the United States of America
SD: standard deviation
ANOVA: one-way analysis of variance
HMG-CoA: 3-Hydroxy-3-methylglutaryl-coenzyme A
ASCVD: atherosclerotic cardio-vascular disease
CVD: cardio-vascular disease
FDA: Food and Drug Administration
GDMT: guideline determined medication therapy
ACEI: angiotensin converting enzyme inhibitor
ARNI: animal rescue need intervention
AMI: acute myocardial infarction
